# The value of predicting restriction of fetal growth and compromise of its wellbeing: Systematic quantitative overviews (meta-analysis) of test accuracy literature

**DOI:** 10.1186/1471-2393-7-3

**Published:** 2007-03-08

**Authors:** Rachel K Morris, Khalid S Khan, Aravinthan Coomarasamy, Stephen C Robson, Jos Kleijnen

**Affiliations:** 1 Academic Department of Obstetrics and Gynaecology, University of Birmingham, Birmingham Women's Hospital, Birmingham, B15 2TG, UK; 2 School of Surgical and Reproductive Sciences, University of Newcastle, Newcastle, NE1 4LP, UK; 3 Kleijnen Systematic Reviews Ltd, York, YO26 6RB, UK

## Abstract

**Background:**

Restriction of fetal growth and compromise of fetal wellbeing remain significant causes of perinatal death and childhood disability. At present, there is a lack of scientific consensus about the best strategies for predicting these conditions before birth. Therefore, there is uncertainty about the best management of pregnant women who might have a growth restricted baby. This is likely to be due to a dearth of clear collated information from individual research studies drawn from different sources on this subject.

**Methods/Design:**

A series of systematic reviews and meta-analyses will be undertaken to determine, among pregnant women, the accuracy of various tests to predict and/or diagnose fetal growth restriction and compromise of fetal wellbeing. We will search Medline, Embase, Cochrane Library, MEDION, citation lists of review articles and eligible primary articles and will contact experts in the field. Independent reviewers will select studies, extract data and assess study quality according to established criteria. Language restrictions will not be applied. Data synthesis will involve meta-analysis (where appropriate), exploration of heterogeneity and publication bias.

**Discussion:**

The project will collate and synthesise the available evidence regarding the value of the tests for predicting restriction of fetal growth and compromise of fetal wellbeing. The systematic overviews will assess the quality of the available evidence, estimate the magnitude of potential benefits, identify those tests with good predictive value and help formulate practice recommendations.

## Background

Restriction of fetal growth and compromise of its wellbeing remain significant causes of perinatal death and childhood disability [1-3]. These babies on reaching adulthood are at greater risk of developing cardiovascular disease, hypertension, and non-insulin dependant diabetes [4,5]. Reliable antenatal identification of the growth-restricted fetus is crucial to judicious allocation of monitoring resources and use of preventative treatment [6] with the prospect of improving perinatal outcome. The variation in the design of research on accuracy of tests for identification of growth restriction and compromise of wellbeing, the scatter of this research across many databases and languages, and the dearth of clear collated up-to-date summaries of this literature contribute to the uncertainty about the best diagnostic and monitoring strategies [7]. A comprehensive systematic review of the literature on all available tests can improve our ability to identify those pregnancies at greatest risk of developing clinically relevant intrapartum and neonatal consequences of impaired fetal growth.

Screening and diagnosis of fetal growth restriction (FGR) and prediction and monitoring for compromise of fetal wellbeing in a clinical setting includes a combination of patients' characteristics, symptoms, physical signs and tests, which form the basis of clinical care. For instance, methods employed to screen for and detect FGR might include obtaining previous history of small babies, recording symphyseal fundal height on a customised growth chart and estimating fetal weight with ultrasound [7]. Similarly, current history of fetal movements, abdominal palpation to assess liquor volume, ultrasound amniotic fluid index, Doppler flow velocimetry and cardiotocography might be used to assess fetal wellbeing [7]. These and other tests outlined in Table 1 will be the focus of our project to systematically review existing research on their accuracy.

**Table 1:** A sample of tests for prediction/diagnosis of fetal growth restriction and/or compromise of fetal wellbeing.

**History**	Clinical risk scoring, abdominal palpation, symphyseal fundal height measurement, fetal movement counting.
**Ultrasound**	Biometry, Doppler, amniotic fluid volume, placental grade, biophysical profile.
**Biochemical Haematological**	e.g. oestriol, human chorionic gonadotrophin, alpha feto protein, pregnancy associated plasma protein (PAPP-A), inhibin A.
**Other tests**	Customised growth charts, cardiotocography, fetal electrocardiogram.

The term FGR (related term IUGR or intrauterine growth retardation) and SGA (small for gestational age) are often used interchangeably, but some times erroneously. Appreciation of the difference between smallness of fetus as a consequence of intra-uterine constraint as opposed to that resulting from normal smallness is central to understanding the meaning of FGR and the accuracy of tests to predict FGR. SGA refers to any fetus that falls below a defined size (e.g. below a particular centile below the population average by a given gestational age). It represents a heterogeneous group comprising of fetuses that have failed to achieve their growth potential (true FGR) as well as fetuses that are constitutionally small due to an inherent low growth potential. Only about half of SGA fetuses (birth weight below 10th centile for gestational age) have FGR. It is FGR that is likely to be associated with childhood disability; normal constitutional smallness is expected to be of no clinical consequence. Hence a good fetal growth test will be expected to accurately identify fetuses that have true FGR, distinguishing FGR from SGA alone.

A distinction has earlier been made between tests that measure fetal size or growth (e.g. biometric tests) and those that assess fetal wellbeing (e.g. biophysical tests) [7]. Tests of wellbeing are aimed at predicting fetal acidaemia, which is perceived, at least in the model of chronic placental failure, to lead ultimately to organ damage and death. Data from fetal blood sampling studies confirm there is a correlation between fetal pH and neurodevelopmental outcome in small fetuses. This implies that the accuracy of tests for FGR need to be assessed separately to those used for assessment of fetal wellbeing, but existing reviews often do not make this distinction.

The reference criteria for confirmation of growth restriction and compromise of wellbeing are different and our project will deal with this issue in a methodologically sound manner. Many tests have been purported to be predict stillbirth, birth hypoxia and neonatal complications [2]. To carefully and strictly define reference standards for our project, we will undertake a systematic review of prognostic studies relating neonatal features of FGR and wellbeing to medium- and long-term outcome, thereby establishing a hierarchy of reference standards. This review will examine how well the available assessments at birth assess the risk of impaired neurodevelopment, [3] educational disadvantage [8] and illnesses (e.g. diabetes mellitus, hypertension) in adult life [9]. We will also use appropriate statistical methods suitable for meta-analysis of studies with various reference standards [10].

Timely prediction of growth restriction and compromise of fetal wellbeing is of essence in antenatal care. Without accurate prediction, clinicians are handicapped. Wrong or delayed prediction puts baby at risk of an adverse outcome whereas correct prediction provides an opportunity to optimise care. If high-risk groups are accurately and efficiently identified, they could benefit from monitoring of wellbeing and appropriate interventions such as steroid administration and timely delivery. However, decision-making is hampered due to lack of precise information on estimates of risk, a situation that can be ameliorated with a comprehensive systematic review of the literature. Research in prevention of complications of growth restriction itself will benefit form identification of high-risk groups with accurate tests as these may be enrolled in clinical trials with an improved likelihood of providing robust evidence of effectiveness.

## Methods/Design

### Objectives

To generate a set of the most accurate tests for predicting restriction of fetal growth and compromise of its wellbeing, systematic reviews (meta-analyses) of test accuracy studies will be conducted with the following objective:

To obtain summary estimates of accuracy of available antenatal tests for predicting restriction of fetal growth and compromise of fetal wellbeing.

### Search Strategy

Literature will be identified using:

• General bibliographic databases including MEDLINE (PubMED) and EMBASE (Ovid)

• Specialist electronic databases: The Cochrane Library (DARE, CCTR), MEDION

• Contact with individual experts and those with an interest in this filed to uncover grey literature

• Contact with manufacturers of tests

• Hand-searching of selected specialist journals

• Checking of reference lists of relevant review articles and papers that will be eligible for inclusion

Two searches will be performed, the first looking at prediction and diagnosis of FGR and the second at compromise of fetal wellbeing. The comprehensive search strategies will aim to find all primary studies reporting in the accuracy of any test (or test combinations) used to predict or diagnose fetal growth restriction and/or compromise of fetal wellbeing. The search for FGR may be viewed as additional file 1: Search strategy for tests to predict/diagnose fetal growth restriction. (other searches are available form the authors on request). This will be achieved by combining search terms relating to FGR/compromise of fetal wellbeing with methodological filters for identification of aetiologic and diagnostic test studies (table 1) [11-13]. All databases will be searched from inception and updated at six monthly intervals. No language restrictions will be applied. A comprehensive database of the literature will be constructed (Reference Manager 11.0).

### Inclusion criteria

Studies will be selected for inclusion in the reviews using the selection criteria based on population, index test, reference standard and study design of interest.

*Population*: Pregnant women any health care setting, any level of risk.

*Diagnostic tests*: Tests will be prioritised on the basis of clinical relevance after consultation with experts in the field. (see table 1)

*Reference standard*: Any measurement of birth weight or nutritional status of newborn performed postnatally.

*Study design*: Observational test accuracy studies (cohorts, case-control prospective) allowing generation of 2 × 2 tables of accuracy. Case series <10 cases and case-control studies defined by reference standard outcome (birth weight measurement) will be excluded, these study designs have been shown to be associated with bias [14].

*Sub-groups*: Severe FGR will be defined as that leading to premature delivery (<37 completed weeks), wherever possible test accuracy for prediction/diagnosis of severe FGR will be assessed.

### Study selection process

Studies will be selected for inclusion in the review in a two-stage process using the selection criteria detailed above. Firstly, the titles and abstracts of the citations in the Reference manager database will be assessed by one reviewer. All papers felt to be relevant will be obtained in full text version. Two reviewers will then independently select the studies, which meet predefined and explicit criteria regarding population, tests, reference standards and study design (defined prior to commencement of the review and individualised for each review). Disagreements will be resolved by consensus or arbitration of a third reviewer.

### Data extraction

For each review a data extraction form will be designed, variation between reviews will mainly be in the information extracted for the performance of the index test. Data will be extracted on: identification of study (first author, year of publication, country of investigation, language of paper); population (healthcare setting, number of participating centres, level of risk assigned by author and clinical data on risk factors, inclusion period); study design (design, data collection, enrolment, completeness of verification); index test (gestation, method of performance of test, intra and interobserver variation, cut-off level); reference standard (incidence, reference standard used, cut-off level, total number of women analysed for results); results (necessary data for construction of 2 × 2 table, all results will be collected for any index test reported at any cut-off level, any measurement of statistical accuracy reported).

The extraction of a study's findings will be conducted in duplicate using the data extraction form. This will help avoid errors in data extraction, disagreements between reviewers will again be resolved by consensus or arbitration of a third reviewer where necessary. Where multiple publications are identified, the most recent and/or complete study will be included only. Data will be entered onto an Excel spreadsheet, after checking of the duplicate extraction forms has resolved any errors.

### Quality assessment

All included manuscripts will be assessed by at least one reviewer for study and reporting quality. Methodological quality is defined as the confidence that the study design, conduct and analysis have minimized biases in addressing the research question, thereby focusing on the internal validity (i.e. the degree to which the results of an observation are correct for the patients being studied), this will be assessed using the QUADAS tool [15,16]. Elements of study design which are likely to have a direct relationship to bias in a test accuracy study will be assessed using the STARD checklist [17].

In the assessment of study quality for the population, consecutive or random recruitment of pregnant women will be considered ideal. Prospective recruitment is considered to introduce less bias than retrospective recruitment. The description of the population will be considered ideal if there is sufficient information about the pregnant women given to assign a level of obstetric risk, ideally this risk level will be stated by the authors in the study's methods. The incidence of FGR will be calculated for each study and used as a check for the author's quantification of the risk category of the population.

Assessing the quality of performance and reporting of the index standard will be individualised for each test enabling the assessment to look at individual aspects of each test that might introduce bias. For the reference standard, any representation of birth weight or nutritional status of the newborn will be considered acceptable. Information will be collected on method of determination of reference standard, execution and blinding.

Ideal study design will be trials or cohort studies, case-control studies will not be included wherever possible, when the number of studies will allowed this, they will however be excluded from meta-analysis as recent papers have shown that this type of study design can affect accuracy [14].

Verification bias will be assessed using a flow chart for each study which will document the number of eligible women for the study, the number of women subjected to the index test, the number of women receiving the reference standard and the number of exclusions, withdrawals and uninterpretable results. Ideal verification will be when all women can be accounted for and the number of eligible women progressing to the reference standard is >90%.

The assessment of quality will be represented by a bar chart. No attempt will be made to apply a quality score as this has been shown to have little validity [18] and quality will not be used as an aspect for inclusion/exclusion of studies from meta-analysis. Wherever possible for each review, an individual assessment will be made of the most important quality items for that test and studies defined as high or low quality. This definition will be used in the sub-group analysis wherever possible.

### Methods of statistical analysis

From the 2 × 2 tables, the true positive rate (sensitivity), false positive rate (1-specificity) and likelihood ratios (LRs) will be calculated for each study along with their 95% confidence intervals (CIs). Where 2 × 2 tables contained zero cells, 0.5 will be added to each cell to enable calculations [19]. In each review, results will be visualised using Forrest plots and ROC plots, extreme values, outliers and threshold phenomena will be explored.

Where appropriate meta-analysis will be used. Pooled summary estimates will be produced in the form of the summary likelihood ratio as this is the measure which is most applicable clinically, in keeping with recommendations from Evidence-based Medicine Groups [20,21]. The likelihood ratios allow estimation of the probability of FGR or neonatal compromise with a specific test result. To generate the practical application of these LRs the post test probability of having the disease will be generated using Bayes' theorem and the following formula: post test probability = likelihood ratio × pre-test probability/[1-pre-test probability × (1-likelihood ratio)]. Estimates of pre-test probability will be made using reports from previous studies and taking into account the risk rates for the population in question. The range of uncertainty will be calculated using the 95% confidence intervals of the likelihood ratios for each test. Either a fixed or random effects model will be used where appropriate following widely published guidelines for their use.

Heterogeneity of results between studies will be assessed graphically by looking at the distribution of sensitivities and specificities in the receiver operating characteristic (ROC) space and LRs as measurement of accuracy size using a Forrest plot. The loglikelihood and X^2 ^test will be used to assess for heterogeneity statistically. Where heterogeneity is not present (X^2 ^> 0.10) the fixed effect pooling method will be used and where possible we will consider the use of the bivariate meta-regression model [22,23]. The model assumes a bivariate normal distribution for the logit-transformed sensitivity and specificity across studies, using a random effects approach for both sensitivity and specificity, allowing for heterogeneity beyond chance due to clinical or methodological differences between studies. In addition, the model acknowledges the difference in precision by which sensitivity and specificity have been measured in each study. The model produces the following results: a random effect estimate of the mean sensitivity and specificity with corresponding 95% CIs, the amount of between-study variation for sensitivity and specificity separately, and the strength and shape of the correlation between sensitivity and specificity. When heterogeneity is present (X^2 ^< 0.10) this will be explored using meta-regression analyses. This will be performed using factors considered to be important beforehand, including:

• Variations in population – high and low risk defined by prevalence of FGR within the population.

• Variations in index test – e.g. type of test parameter, cut-off used

• Variations in reference standard – test used, threshold used

• Study quality

• Study design – cohort studies only.

Analysis for assessing the risk of publication bias will be carried out by producing funnel plots [24] of accuracy estimates against corresponding variances. When no publication bias is present the plots will be shaped like a funnel because studies of smaller size are expected to have increased variation in the estimates of accuracy. The bigger the study variance, the lower the weighting of the study and the less information it provides. This means that in addition to small sample size of included primary studies, those studies reporting very high accuracy will also have a relatively big variance and thus be weighted less. There is however debate about methods for this issue in diagnostic reviews [25].

Data synthesises were performed using Meta-DiSc version 1.4[26], SATA version 9.0 and SAS version 8.2.

## Data description

For each test, information on individual studies was summarised as follows:

• **Table with methodological and reporting characteristics of included studies**.

The table states the number of women tested in each study, the incidence of fetal growth restriction (based on the number of analysed cases divided by the total number of women at baseline (cohort studies and nested case-control studies)) and maternal age (given as mean (± SD) for the whole group unless otherwise stated).

• **Summary of quality and reporting items of the included studies**.

Results were presented as 100% stacked bars, where figures in the stacks represent the number of studies. The item 'study design' is stated in the table with quality and reporting characteristics.

• **Forest plots of sensitivities (%) and specificities (%) and 95% CIs**.

Studies are ranked according to decreasing specificity (within subgroups). Numbers of women analysed are tp/(tp+fn) for sensitivity and tn/(fp+tn) for specificity.

• **ROC plot**.

Estimates of predictive accuracy from individual studies are shown (separate for subgroups if appropriate) and where possible a summary ROC curve was drawn. In the summary ROC plots the vertical axis shows sensitivity, while the horizontal axis shows one-minus-specificity.

• **Table with subgroup analyses**. (If applicable.)

Significance level p < 0.10.

## Discussion

The methodology of diagnostic systematic reviews is rapidly developing with the recent development of guidelines on conducting primary studies [27] and their reporting [17] and validation of quality assessment tools [16]. This has lead to studies investigating the affect of study design and conduct on diagnostic accuracy [14,28]. The bivariate method is a statistical method designed specifically for diagnostic data and its use will allow us to estimate the degree of correlation between sensitivity and specificity and thus provide information about heterogeneity and the possibility if an implicit threshold [22,29].

This project will utilise all the recent developments in methodology of systematic reviews of diagnostic tests as well as developments in statistical analysis. The results of the reviews will help produce a set of accurate tests to predict/diagnose fetal growth restriction and compromise of its wellbeing that can be incorporated into guidelines for clinical practice.

One of the anticipated problems with this project is the lack of knowledge surrounding measurements of weight at birth and nutritional measures and there relation to neonatal morbidity and mortality and future childhood disability [30][31][32][33][34]. We will thus also aim to undertake a systematic review of prognostic studies relating neonatal features to outcome (e.g. childhood disability, neurodevelopmental outcome) thereby establishing a hierarchy of reference standards. This will not only help us with the interpretation of the results from our diagnostic accuracy reviews but also help guide future primary research. Results will be available through 2007–08.

## Competing interests

The author(s) declare that they have no competing interests.

## Authors' contributions

RKM developed the protocol, developed the searches and data management, will participate in selection, inclusion, quality assessment and data extraction of papers for the reviews, will perform statistical analysis. KSK led the grant application secured from Wellbeing of Women charity, developed the protocol and will advise RKM in all of her work as the general project supervisor. SCR developed the protocol, will provide clinical expertise and review of manuscripts for publication. AC helped develop the protocol. JK helped develop the protocol, will provide advice on methodology and statistical analysis and review of manuscripts for publication. All authors read and approved the final manuscript.

## Pre-publication history

The pre-publication history for this paper can be accessed here:



## Supplementary Material

Additional File 1 Search strategy for tests to predict/diagnose fetal growth restriction. Word document detailing search strategy for Medline, Embase and Cochrane library used in systematic reviews of diagnostic accuracy of tests to predict/diagnose fetal growth restriction. Click here for file
