# Supplementary material for: The value of predicting restriction of fetal growth and compromise of its wellbeing: Systematic quantitative overviews (meta-analysis) of test accuracy literature
Source: BMC Pregnancy Childbirth. 2007 Mar 8;7:3. doi: 10.1186/1471-2393-7-3 (PMC1828066; doi:10.1186/1471-2393-7-3)
Supplement: Additional File 1 — Search strategy for tests to predict/diagnose fetal growth restriction. Word document detailing search strategy for Medline, Embase and Cochrane library used in systematic reviews of diagnostic accuracy of tests to predict/diagnose fetal growth restriction. [file 1471-2393-7-3-S1.doc]

**Search strategy for tests for prediction/diagnose of fetal growth restriction.**

**PUBMED/MEDLINE:**

**Set 1**:

Growth restriction

1. (("Small-for-Gestational Age") OR (Small-for-Gestational Age) OR (lbw) OR (small for gestational age) OR (sga) OR (small for date*) OR (small for gestation*) OR (fgr) OR (iugr) OR (intrauterine growth retard*) OR (intrauterine growth restrict*) OR (fetal growth retard*) OR (fetal growth restrict*) OR (growth restrict*) OR (growth retard*) OR ("Placental Insufficiency"[MeSH]) OR ("Fetal Growth Retardation"[MeSH]) OR ("Infant, Low Birth Weight"[MeSH])) OR (low birth weight)

**Set 2**:

Pregnancy

2. ("Pregnant Women"[MeSH] OR "Pregnancy"[MeSH] OR "Pregnancy Outcome"[MeSH]) OR (pregnan*)

**Set 3**:

Diagnosis filter

3. Sensitivity and Specificity[MeSH] OR predict* OR diagnose* OR diagnosi* OR diagnost* OR accura*

**Set 4:**

Aetiology-filter

4. ((((("cohort studies"[mh] OR "case-control studies"[MeSH Terms]) OR "risk"[mh]) OR "epidemiologic factors"[MeSH Terms]) OR ("odds"[tw] AND "ratio*"[tw])) OR ("relative"[tw] AND "risk"[tw])) OR ("case"[tw] AND "control*"[tw])

5. ((#1 AND #2) AND (#3 OR #4))

**OVID/EMBASE**:

1 exp pregnancy/

2 exp Pregnant Woman/

3 pregnancy outcome.mp.

4 pregnan$.mp. [mp=title, abstract, subject headings, heading word, drug trade name, original title, device manufacturer, drug manufacturer name]

5 pregnant wom$.mp. [mp=title, abstract, subject headings, heading word, drug trade name, original title, device manufacturer, drug manufacturer name]

6 1 or 2 or 3 or 4 or 5

7 exp Fetus Growth/

8 low birth weight.mp. or exp Low Birth Weight/

9 exp Intrauterine Growth Retardation/

10 Intrauterine Growth Retard$.mp.[mp=title, abstract, subject headings, heading word, drug trade name, original title, device manufacturer, drug manufacturer name]

11 Growth Retard$.mp. [mp=title, abstract, subject headings, heading word,

drug trade name, original title, device manufacturer, drug manufacturer

name]

12 Fetal Growth Retard$.mp. [mp=title, abstract, subject headings, heading word, drug trade name, original title, device manufacturer, drug manufacturer name]

13 intrauterine growth restrict$.mp. [mp=title, abstract, subject headings, heading word, drug trade name, original title, device manufacturer, drug manufacturer name]

14 fetal growth restrict$.mp. [mp=title, abstract, subject headings, heading word, drug trade name, original title, device manufacturer, drug manufacturer name]

15 growth restrict$.mp. [mp=title, abstract, subject headings, heading word, drug trade name, original title, device manufacturer, drug manufacturer name]

16 exp Small for Date Infant/

17 Small for gestational age.mp.[mp=title, abstract, subject headings, heading word, drug trade name, original title, device manufacturer, drug manufacturer name]

18 Small for date$.mp. [mp=title, abstract, subject headings, heading word,

drug trade name, original title, device manufacturer, drug manufacturer

name]

19 Small for gestation$.mp. [mp=title, abstract, subject headings, heading word, drug trade name, original title, device manufacturer, drug manufacturer name]

20 fgr.mp. [mp=title, abstract, subject headings, heading word, drug trade name, original title, device manufacturer, drug manufacturer name]

21 iugr.mp. [mp=title, abstract, subject headings, heading word, drug trade name,

original title, device manufacturer, drug manufacturer name]

22 sga.mp. [mp=title, abstract, subject headings, heading word, drug trade

name, original title, device manufacturer, drug manufacturer name]

23 exp Placenta Insufficiency/

24 7 or 8 or 9 or 10 or 11 or 12 or 13 or 14 or 15 or 16 or 17 or 18 or 19 or 20 or 21 or 22 or 23

25 6 and 24

26 (sensitiv$ or detect$ or accura$ or specific$ or reliab$ or positive or negative or diagnos$).mp. or di.fs.

27 cohort analysis/

28 exp risk/

29 (odds$ adj ratio$).mp.

30 (relative adj risk).mp.

31 case control study/

32 (case$ adj control$).mp.

33 (causa$ or predispos$).mp.

34 25 or 26 or 27 or 28 or 29 or 30 or 31

35 26 or 34

36 25 and 35

**COCHRANE LIBRARY:**

#1 small for gestational age in All fields in all products

#2 sga in All fields in all products

#3 small for date in All Fields in all products

#4 fgr in All fields in all products

#5 lbw in All fields in all products

#6 iugr in All fields in all products

#7 intrauterine growth retard* in All fields in all products

#8 fetal growth retardation in All fields in all products

#9 fetal growth retard* in All fields in all products

#10 growth restrict* in All fields in all products

#11 growth retard* in All fields in all products

#12 low birth weight in All fields in all products

#13 MeSH descriptor Placental Insufficiency explode all trees in MeSH products

#14 placental insufficiency in All fields in all products

#15 (#1 or #2 or #3 or #4 or #5 or #6 or #7 or #8 or #9 or #10 or #11 or #12 or #13 or #14)

#16 PREGNANCY in All fields in all products

#17 MeSH descriptor Pregnant women explode all trees in MeSH products

#18 MeSH descriptor Pregnancy explode all trees in MeSH products

#19 MeSH descriptor Pregnancy outcome explode all trees in MeSH products

#20 (#16 or #17 or #18 or #19)

#21 predict* or diagnose* or diagnosi* or diagnost* or accura* in All fields in all products

#22 MeSH descriptor Sensitivity and Specificity explode all trees in MeSH products

#23 (#21 or #22)

#24 MeSH descriptor Cohort studies explode all trees in MeSH products

#25 MeSH descriptor Case-control studies explode all trees in MeSH products

#26 MeSH descriptor Risk explode all trees in MeSH products

#27 MeSH descriptor Epidemiologic Factors explode all trees in MeSH products

#28 (odds AND ratio) OR (relative AND risk) OR (case AND control) in All fields in all products

#29 (#24 or #25 or #26 or #27 or #28)

#30 (#15 and #20)

#31 (#23 or #29)

#32 (#30 and #31)
